# Phase Synchronization in Electroencephalographic Recordings Prognosticates Outcome in Paediatric Coma

**DOI:** 10.1371/journal.pone.0094942

**Published:** 2014-04-21

**Authors:** Vera Nenadovic, Jose Luis Perez Velazquez, James Saunders Hutchison

**Affiliations:** 1 Division of Neurology Sick Kids, Toronto, Ontario, Canada; 2 Brain and Mental Health, Toronto, Ontario, Canada; 3 Department of Critical Care Medicine Sick Kids, Toronto, Ontario, Canada; 4 Institute of Medical Science, University of Toronto, Toronto, Ontario, Canada; University of British Columbia, Canada

## Abstract

Brain injury from trauma, cardiac arrest or stroke is the most important cause of death and acquired disability in the paediatric population. Due to the lifetime impact of brain injury, there is a need for methods to stratify patient risk and ultimately predict outcome. Early prognosis is fundamental to the implementation of interventions to improve recovery, but no clinical model as yet exists. Healthy physiology is associated with a relative high variability of physiologic signals in organ systems. This was first evaluated in heart rate variability research. Brain variability can be quantified through electroencephalographic (EEG) phase synchrony. We hypothesised that variability in brain signals from EEG recordings would correlate with patient outcome after brain injury. Lower variability in EEG phase synchronization, would be associated with poor patient prognosis. A retrospective study, spanning 10 years (2000–2010) analysed the scalp EEGs of children aged 1 month to 17 years in coma (Glasgow Coma Scale, GCS, <8) admitted to the paediatric critical care unit (PCCU) following brain injury from TBI, cardiac arrest or stroke. Phase synchrony of the EEGs was evaluated using the Hilbert transform and the variability of the phase synchrony calculated. Outcome was evaluated using the 6 point Paediatric Performance Category Score (PCPC) based on chart review at the time of hospital discharge. Outcome was dichotomized to good outcome (PCPC score 1 to 3) and poor outcome (PCPC score 4 to 6). Children who had a poor outcome following brain injury secondary to cardiac arrest, TBI or stroke, had a higher magnitude of synchrony (R index), a lower spatial complexity of the synchrony patterns and a lower temporal variability of the synchrony index values at 15 Hz when compared to those patients with a good outcome.

## Introduction

Brain injury is the most important cause of death and acquired disability in the paediatric population [1]. Traumatic injury is the most common, followed by ischemic injury from cardiac arrest and stroke [1]–[3]. The neurological sequelae of brain injury have implications for the child’s cognitive and social development [4], [5]. Traumatic brain injury and ischemic brain injury are not discrete events but disease processes that evolve over time and the outcome of which can be improved with accurate and early diagnosis [6]. Thus there is a need for methods to stratify patient risk and ultimately predict outcome [7]. The ideal method for monitoring brain function and predicting outcome would be non-invasive, portable and accurate. To reflect changing brain dynamics it would be performed in real-time. This could improve the type of treatment and its timeliness, as well as the prognostication of outcome [8]. The latter would help mitigate the distress, anxiety and the post-traumatic stress that can be experienced by families of patients as they wait and see what will happen to their child [9].

Admission to PCCU is required for severe brain injury when life support is required [10]. Clinicians in the paediatric critical care unit (PCCU) frequently manage children in coma following severe brain injury. Coma is defined as absence of awareness of self and the external environment and measured by a Glasgow Coma Score (GCS) <8. It can result from brain injury from a variety of aetiologies including cardiac arrest, trauma, stroke and infection [11]. Assessing brain function in comatose patients in the critical care unit is difficult due to the nature of coma and medical intervention such as sedation and the use of paralytic agents [12]. In brain injury, the Glasgow Coma Scale (GCS) has been the gold standard for evaluating coma in both children and adults [13], [14]. However, assessing brain function in comatose patients in the critical care unit is difficult [12]. Clinical neurological examination provides limited information in the comatose, muscle-relaxed, intubated patient [12], [15].

Without accurate monitoring and assessment of brain function early prognostication of outcome is extremely difficult [16]. However, clinicians still rely on combinations of neuroimaging, clinical scores, clinical experience, neuroelectrophysiology and biomarkers for estimating prognosis for the purposes of: directing treatment, allocating resources and informing parents and caregivers of potential outcomes [17]–[20]. Noninvasive brain monitoring in PCCU consists of many modalities utilized alone or in combination, but except for electroencephalography (EEG), they are all static providing information at one time point [7]. Brain activity is dynamic.

Neuroimaging is commonly performed: magnetic resonance imaging (MRI), computed tomography (CT), and ultrasound (in infants with an open fontanelle). MRI requires transport from the relative stability of the PCCU to the imaging suite, as does CT for those facilities without portable CT [21]. Moreover, the CT scan exposes the patient to radiation and this has recently become a concern due to the associated increase risk of cancer in children [22]. Functional MRI (fMRI) is a useful tool in the cooperative subject who can perform the task required. Apart from a few adult studies of patients in a minimally conscious state (not coma) fMRI is generally not possible in a comatose patient, the very young or in any patient who could not cooperate with demands [23].

Electrophysiological studies provide a noninvasive, bedside modality for evaluating neurological function. Initial and serial evoked potentials (somatosensory, visual and auditory) have been used for both monitoring and prognostication in traumatic brain injury and hypoxic-ischemic encephalopathy [24]–[26]. To date, the somatosensory evoked potentials have had consistent prognostic utility in anoxic brain injury, but not in trauma. In many studies the evoked potentials have been used in conjunction with electroencephalography (EEG) to assess brain function and predict outcome [27]–[29].

Electroencephalography (EEG) has been used with some success as a prognostic tool, with researchers developing scores based on the raw recordings [30]. All of these studies show some utility of neuroelectrophysiology in monitoring brain function and predicting outcome, but again, none have been universally adopted. This is due to the heterogeneity of presenting pathology and the changing anatomic and synaptic configuration of the developing brain in children [31], [32]. Electroencephalography can assess the brain’s dynamic activity. It is resource intense, requiring specialist interpretation of the waveforms [12]. Information on brain function changes is often not immediate as the neurologist reading the recordings may be doing so remotely without direct access to the patient and may be reviewing changes retrospectively.

Accurately predicting outcome would enable clinicians in paediatric critical care to anticipate consequences, thereby focusing treatment and rehabilitation and potentially improving long-term outcome. Early prognosis is fundamental to the implementation of interventions to improve recovery and there is an increasing interest in detecting “biomarkers” for brain injury [8], [17]. Most of these are sought at the molecular level, but the complexity of the brain likely precludes a simple model or a single diagnostic tool for accurate prediction. Since prediction models have been examined in children utilizing combinations of clinical parameters, electrophysiology and neuroimaging, and still no practical model exists, there is an opportunity for a new biomarker to be evaluated [18], [33].

In our previous work in critically ill children post traumatic brain injury (TBI), we discovered a promising biomarker that focused on one aspect of the property of brain complexity. We evaluated the correlation between brain variability using electroencephalographic (EEG) recordings and outcome [34]. As our initial study focused solely on coma in TBI, for our biomarker to be of clinical value in the PCCU setting, we chose to validate it in other aetiologies of brain injury leading to coma.

Healthy physiology is associated with a relative high variability in physiologic signals [35]–[37]. This phenomenon is well known in the study of heart rate variability. Heart rate variability has been studied since the 1970’s as a biomarker and has been used to both assess cardiac function and as an early predictor of neonatal sepsis [38]–[40]. Variability in physiological signals provides information than is available on visible inspection of the raw signal [41], [42]. The EEG is another noninvasively acquired physiological signal whose variability can be quantified. In this study we build on our initial study in TBI to evaluate brain variability in brain injury of different aetiologies using EEG.

Basic neurophysiological activity is altered following brain injury. In the initial phases post-injury, cell hyperexcitability occurs [43]. The EEG recordings reveal a generalized slowing of brain frequencies to the delta and theta ranges [43], [44]. Time dependent alterations in synaptic function following cortical injury and structural damage with subsequent cell reorganization have been well described in the 1990s [45], [46]. Electrophysiological analysis of patients with cerebral trauma and concussion was first reported in the 1970’s [44], [47]. The coherence of electroencephalogram signals was evaluated a decade later in a few studies and thought to reflect neuroanatomical damage [48]. This method was used to extract information on cortico-cortical associations and functional connectivity that were not otherwise visible on the raw EEG recording-[48]–[50].

Recently, EEG phase synchrony, a method related to coherence, has been used for the first time to evaluate brain function after traumatic brain injury (TBI) in adults and in children [34], [51]. Both studies showed changes in EEG phase synchrony in the days following trauma. Our study in addition to the phase synchrony changes, showed that the variability of the EEG phase synchrony increased as children emerged from coma, despite the absence of visible improvement in the EEG recording. The EEG phase synchrony and variability measures provided information on brain function that was not available by other means such as visual inspection [34], [52], [53]. In our study these measures were able to differentiate between patients with good outcome versus poor neurological outcome, the latter having higher magnitudes of EEG phase synchrony and lower variability values [34].

In our current study, we validated those findings in a fully powered retrospective study of children in coma due to brain injury from traumatic and hypoxic (cardiac arrest) or ischemic events (stroke). We hypothesised that variability in brain signals could serve in the prognostication of patients after brain injury. Lower fluctuations in brain synchronization, derived from EEG recordings, will be associated with poor patient prognosis. We thus presented evidence for lower spatio-temporal variability associated with poor outcome in children after acute brain injury, and proposed that the evaluation of variability in brain signals can have a valuable impact in the prognosis of brain injury in the acute phase. We also evaluated the phase synchrony and spatio-temporal variability of specific EEG electrodes. In the resource and time constrained critical care environment we were looking for an abbreviated montage of EEG electrodes that could yield prognostic information.

## Materials and Methods

We performed a retrospective study, spanning 10 years (2000–2010) of children aged 1 month to 17 years who met the following inclusion criteria: were in coma (Glasgow Coma Scale, GCS, <8); had been admitted to the critical care unit following brain injury from TBI, cardiac arrest or stroke and had had an EEG recording. The REB (Research Ethics Board) at the Hospital for Sick Children approved our application for waived consent as our study was a retrospective chart review and our findings would not impact patient outcome (REB File #1000004603).

### Outcome Measure

The outcome measure used was the Paediatric Cerebral Performance Category score (PCPC score). This is a validated six point outcome measure for critically ill children [54]–[56]. A PCPC score of 1 indicates normal function for age, 2 is mild disability, 3 is moderate disability, 4 is severe disability, 5 is persistent vegetative state and 6 is death. The outcome was dichotomized into 2 categories: good outcome (PCPC score 1–3) and bad outcome (PCPC score 4–6). As this was a retrospective study, outcome at the time of hospital discharge was assessed through retrospective chart review. The patient assessment information contained in the physiotherapy and occupational therapy notes closest to the time of discharge was used to calculate the PCPC score. If that note was not available, the physician discharge summary note was used. The outcome measure was evaluated while the EEG variables were calculated by the Matlab programs so as not to bias the chart review.

### EEG Acquisition

Scalp EEGs for patients were acquired by accredited EEG technologists, using the international 10–20 montage system. The recordings consisted of 19 channels, referenced to an electrode adjacent to the midline parietal (PZ) channel which was labelled PZ’ (PZ prime). The EEGs were 30 minutes in length. All EEGs acquired with either the XLTek (sampling rate 250 Hz) or Stellate Harmonie (sampling rate 500 Hz) systems as the hospital changed the EEG recording equipment in the ten year interval of this retrospective study. All recordings had a bandpass of 1 to 70 Hz with a 60 Hz notch filter. Acceptable impedances in the intensive care setting were 100 to 5000 Ohms. All patients had electromyographic (EMG) electrodes to record muscle movement, and electrocardiogram (EKG) leads. As all patients were comatose and intubated, the EEGs were acquired with the child in a recumbent position with the head in a midline position. Whenever possible no nursing interventions occurred during the 30 minute acquisition time. When patient intervention was required, this was documented by the EEG technologist and those epochs were not extracted for analysis.

### Frequency and Epoch Selection

In the paediatric critical care environment there are multiple variables that impact EEG frequency. Many medications that affect electroencephalography recordings are commonly used in the treatment of critically ill children. Phenytoin is frequently used post traumatic brain injury as seizure prophylaxis [57]. The background alpha frequency (8 to 12 Hz) is slowed by phenytoin [58]. Benzodiazepines such as lorazepam and midazolam are used to treat seizures and also to provide patient sedation. An increase in the higher beta frequencies (18 to 25 Hz) is seen in electroencephalography recordings with use of benzodiazepines [40].

Electroencephalographic recordings change with respect to the background frequency as children mature, increasing from the theta (4 to 7 Hz) to the alpha (8 to 12 Hz) bandwidth [40]. Patient ages in our PCCU range from newborn to 17 years old, representing a wide range of EEG developmental characteristics. Inotrope infusions such as dopamine and norepinephrine used for many of the cardiac arrest patients have also been shown to increase EEG activity in animal models [59], [60].

The heterogeneity of the medications used within each of the diagnostic groups and the number of patients that fit inclusion criteria were such that there would not be sufficient power to stratify either by medication class (eg. Benzodiazepines, opioids, phenytoin) or combination of medications. The medication confounder was addressed by the choice of bandwidths after review of the literature and consultation with 3 paediatric neurology colleagues with experience in neurocritical care. For the EEG phase synchrony and spatio-temporal variability to be of practical use in the critical care setting, it would have to be applicable across diagnostic categories and medications. The chosen bandwidths: the delta (3 Hz) and lower beta (15 Hz) are the least affected by most of the medications used in paediatric critical care, however not completely unaffected. The Matlab program for the Hilbert transform requires a±2 Hz bandwidth around each central frequency (3 Hz±2 Hz; 15 Hz±2 Hz).

The EEGs were read by the first author and then verified by an EEG certified paediatric neurologist. The EEGs were read for the purposes of identifying encephalopathic waves, for the presence of seizures, for any sleep features and for the presence of artefact. Four 10 second epochs were selected from each recording. The epochs selected were artefact free and represented the general EEG background activity. Selected epochs did not include seizure activity or sleep features, as these phenomena could increase the overall phase synchrony. Care was taken to obtain artefact-free epochs because the ICU is an environment with multiple sources of electrical interference and potential artefact sources in the form of IV infusions, ventilators, air mattress inflation and deflation and patient and care provider movement at the bedside. The epochs were exported as text files for analysis with Matlab.

### EEG Phase Synchrony Calculation

After selecting artefact-free epochs, the first processing of the EEG signals was to remove the common reference electrode used in scalp EEG that significantly alters measures of coherence [61]. For this purpose, a Laplacian derivation was performed. In addition to the removal of the common reference, the Laplacian derivation, which approximates a reference-free signal, has the advantage of attenuating volume conduction effects [62]–[65].

**Figure 1:**
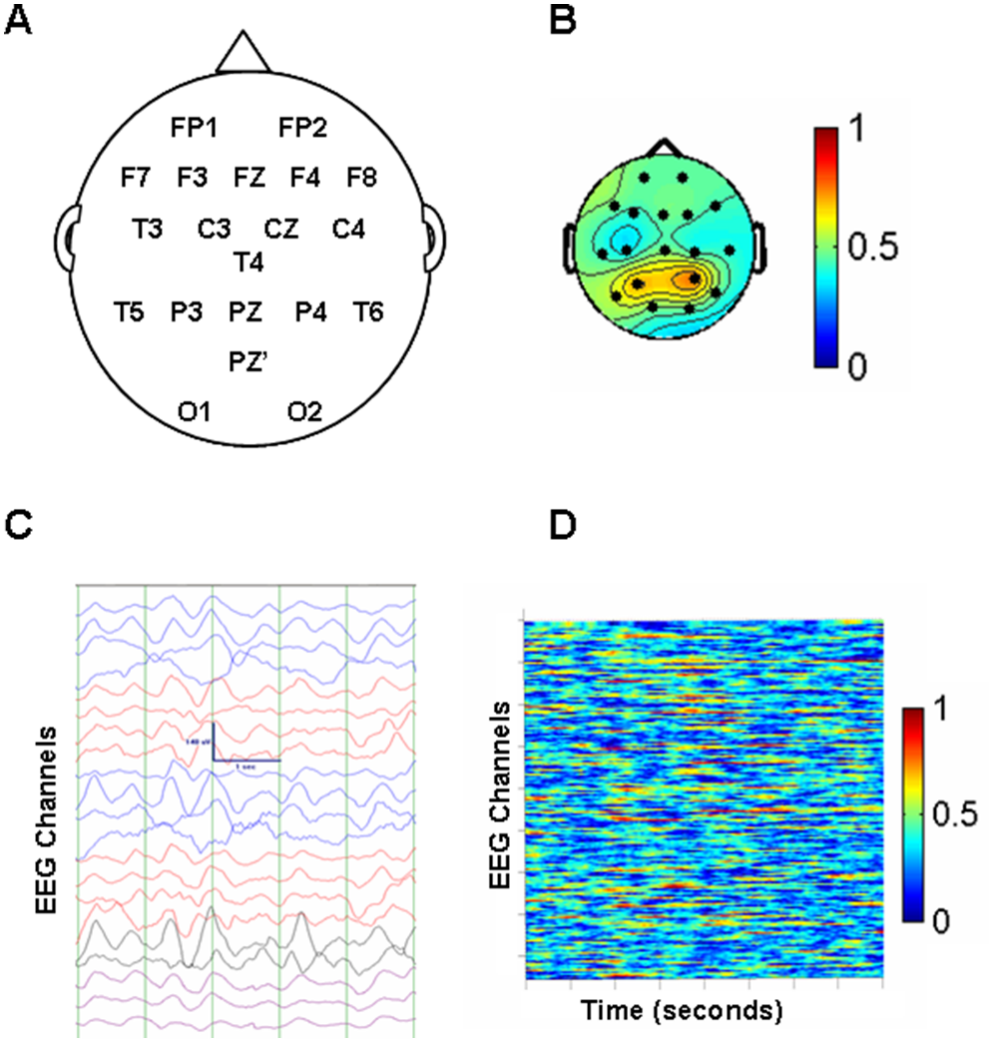
EEG signal analysis. Figure 1 demonstrates the conversion from the raw EEG signal to phase synchrony. Panel A shows the placement of the 19 scalp electrodes, with Pz’ (Pz prime) as the reference electrode. Panel C, below it, shows a typical, slow EEG recording (∼1 Hz) of a patient in coma. The green vertical lines represent 1 second. Panel B shows one method of representing phase synchrony values. The headplot corresponds to the electrode placement in panel A, where each black dot represents one of the 19 electrodes (the reference electrode is not shown). The phase synchrony values from 0 to 1 are the average value for the 10 second recording. The values at each electrode are colour coded with an R value = 0 for the 10 seconds represented as blue to a maximal synchrony, R = 1, represented as red. Panel D corresponds to an excerpt of the 10 second recording in panel C. Panel C is a 5 second excerpt of the digital EEG recording, where the rows from top to bottom represent the 19 electrodes, and the time in seconds is on the x axis. The phase synchrony values (0, blue to 1, red) are calculated for each second of the recording and are mapped over the 8 second time epoch.

Next, signals were band-passed with an order 100 Constrained Least Square Finite Impulse Response filter (FIRCLS) (f±2Hz) prior to the extraction of the instantaneous phases using the Hilbert Transform. The Hilbert Transform is particularly useful for analyzing the electroencephalogram whose waveforms are nonstationary and have multiple frequencies that change over time by extracting the instantaneous phase of the signal [66], [67]. Phase synchronization is then calculated as the degree of phase locking between two channels using the circular variance of the phase difference distribution 
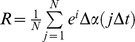
 where |.| denotes the vector magnitude and N is the number of data points that are being considered, and 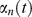
denotes the instantaneous phase of signal n so that the phase difference is computed as: 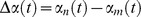. The resulting index R, computed in this manner, quantifies the degree of 1∶1 phase locking in a specific time window [68]. A time window of 1 second was used in our study. The results can be represented as an R value for each of the 171 non-repeating combinations of channel pairs among the 19 electrodes. As an example, the R value for electrode 1 (F7-left frontal region) with electrode 2 (T3-left temporal region) is the same as that computed for electrode 2 with electrode 1, therefore the total number of non-repeating channel pair combinations is: 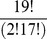 The R values can then be averaged for each of the channels. For example, the average of the 18 R values of electrode 1, F7, with the remaining 18 electrodes, becomes the R value for electrode F7. The 19 values of the synchrony index R can be mapped on a head plot as was shown in figure 1, panel B.

### Spatial Variability of the Synchronization Pattern

Our method that we termed spatial complexity (SC) measures how tightly the phase synchronization (R) values cluster around a single mean value [69]. The algorithm for calculating SC produces an output, *E_j_* that is dependent on the phase synchrony index, R, as denoted by: 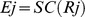. It measures how predictable the synchrony indices are for each channel using the spatial information from the neighbouring values. The R index is first computed as described above from the instantaneous phase differences for each of the 171 pair of channels and an average for each channel is computed. Since there are 19 of such maps each one can be identified as Mj (j = 1,…,19). On each map Mj we compute a spatial complexity index and assign it to the respective j channel.

As previously described in the original article by Garcia Dominguez et al., 2007, an iterative process is used [49]. The R index is calculated for each pivot channel, the pivot channel is then removed and the estimated R index for that channel is calculated from the remaining channels. The SC is the sum of the difference between the estimated and actual values.



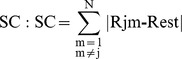



Homogeneous patterns of phase synchrony will have little error with the estimated value and actual being similar, resulting in a lower SC value. In contrast, heterogeneous patterns with variable R values at each channel will result in a higher error and a higher SC value.

### Temporal Variability

The spatial complexity evaluates the variability or fluctuations in the EEG phase synchrony across brain regions at one time point; another measure of the variability of the EEG phase synchrony evaluates fluctuations over the length of the specified time epoch. The temporal variability of the EEG phase synchronization is the variance of the time series of R values. It was calculated as the mean value of the square of the derivative of the time series. Temporal variability was calculated for each epoch for each EEG for each patient. The four epochs were then averaged resulting in a global temporal variability measure for each patient or subject.

### Statistical Analysis

Sample size was calculated based on our study of the temporal variability of the EEG phase synchrony in critically ill children post TBI [35]. The means and standard deviations of the two outcome groups (good outcome = PCPC score 1 to 3; poor outcome = PCPC score 4 to 6) were used with an alpha set at 0.05 and power = 0.80. This power calculation resulted in a minimum sample of 24 patients per outcome group.

Statistical analyses began with descriptive statistics: means, standard deviations and ranges for diagnostic and outcome groups. As the outcome groups were unbalanced, the Welch-Satterthwaite t-test, which does not assume equal variances was used to evaluate the difference between them. The Chi square was first employed to characterize the patients by diagnostic category. Logistic regression was used to evaluate the effect of the variables: age, gender, diagnostic category, R index, spatial complexity and temporal variability at both 3 and 15 Hz on outcome. Statistical significance was set at p = 0.05. Analysis of Variance (ANOVA) was used to evaluate the EEG phase synchrony, spatial complexity and temporal variability of the EEG electrodes with respect to outcome. The Bonferroni correction was used for multiple comparisons. The effect size of statistically significant variables was calculated as Cohen’s d coefficient [70]. All statistical analyses were performed using SAS.

## Results

### Group Demographics

There were 84 children who met the criteria for inclusion, having had a brain injury from cardiac arrest, traumatic brain injury or stroke and having had a 19 channel EEG. They were grouped as follows: Cardiac arrest, n = 30; TBI, n = 35 and stroke, n = 19. Of the total patients (n = 84) the majority, 51/84 (60.7%), had a poor outcome. In contrast only 33/84 (39.3%) had a good outcome. Of the 51 patients who had a poor outcome, 30 of these (58.8%) died, therefore they had a PCPC score = 6. There was only 1 patient of the 51 patients with poor outcome in a persistent vegetative state at the time of hospital discharge (PCPC score = 5).

The groups were analyzed with respect to age, gender and outcome. The age demographics are summarized in Table 1. The cardiac arrest and stroke groups had more patients who experienced a poor outcome (PCPC score 4 to 6) compared to those patients in the TBI group. Chi square analysis showed that this was statistically significant (p = 0.002). Despite the wide age ranges, logistic regression found that age was not a statistically significant variable with respect to outcome for any of the diagnostic groups. There were more males than females in each diagnostic category [59 (70.2%), male; 25 (29.8%), female], but this was not statistically significant between groups.

**Table 1:** Patient demographics.

Diagnosis	N	%	Mean Age (Years)	Standard Deviation (Years)	Range (Years)
Cardiac arrest	30	35.7	5.7	6.1	0.02–17
TBI	35	41.7	7.3	5.3	0.75–17
Stroke	19	22.6	7.8	6.2	0.75–17
Total	84	100.0	6.8	5.8	0.02–17

Table 1 shows the number, mean age, standard deviation and age range for each of the 3 diagnostic categories and the total sample. The patients who had suffered cardiac arrest were the youngest of the three diagnostic categories. However analysis of variance (ANOVA) showed that there is no statistically significant difference between the groups with respect to age: F = 0.95, p = 0.39.

### EEG Phase Synchrony and Spatio-temporal Variability Measures

Patients with poor outcome (PCPC score 4–6) had higher EEG phase synchrony than those with good outcome (PCPC score 1–3) at both bandwidths. This difference was statistically significant only in the 15 Hz bandwidth. These results are summarized in Table 2. Patients with poor outcome also had lower spatio-temporal variability compared to patients with good outcome. Again this was statistically significant in the 15 Hz bandwidth. Logistic regression demonstrated that the differences in the measures were not a function of the diagnostic category.

**Table 2:** Mean R indices, spatio-temporal variability and outcome.

Measure (Mean Values)	PCPC 1–3 Good Outcome	PCPC 4–6 Poor Outcome	T-Test P value
R value–3 Hz	0.591±0.082	0.613±0.098	0.5
R value–15Hz	0.412±0.152	0.491±0.217	0.03
Spatial Complexity–3 Hz	0.163±0.042	0.157±0.048	0.64
Spatial Complexity–15 Hz	0.175±0.019	0.158±0.032	0.02
Temporal Variability–3 Hz	0.00198±0.0004	0.00199±0.0004	0.78
Temporal Variability–15 Hz	0.00220±0.0005	0.00196±0.0007	0.03

Table 2 presents the mean R indices and spatio-temporal variability measures for the 2 outcome groups. The values in the table are the means and standard deviations for each of the 3 parameters: the R index, the spatial complexity and temporal variability values for the 2 outcome groups, good and poor, at both frequencies (3 and 15 Hz). The p value of the Student t-test is provided for each comparison.

The effect size, d coefficient, of the statistically significant variables: phase synchrony (R), spatial complexity and temporal variability at 15 Hz was calculated. The temporal variability had the least overlap (d = 2.8, 8% overlap) compared to that of the phase synchrony (d = 0.5, 66% overlap) and the spatial complexity (d = 0.63, 62% overlap).

### Analysis by EEG Electrodes

Analysis of the data with respect to outcome and EEG electrodes demonstrated that some electrodes showed statistically significant differences in the EEG synchrony, spatial complexity and temporal variability values of patients with poor outcome, compared to those with good outcome. Post hoc analysis revealed that spatial complexity of phase synchrony (R index) associated with the parietal electrodes at 15 Hz, was significantly higher in patients with good outcome. The frontal electrodes also showed consistently higher values of spatial complexity for patients with good outcome, though of the 6 frontal electrodes, only 2 had statistically significant differences at the 15 Hz bandwidth. A summary of the significant differences associated with the parietal and frontal electrodes demonstrated that the mean spatial complexity is higher in the frontal and parietal electrodes in patients with good outcome measured by PCPC = 1–3. These results are presented in Table 3. When all EEG electrodes were examined and the Bonferroni correction for multiple comparisons was used, only those electrodes with a p value of <0.003 were statistically significant.

**Table 3:** Spatial complexity by EEG electrodes.

EEG Channel	Good Outcome PCPC 1–3	Poor Outcome PCPC 4–6	T-test p value
F3–15 Hz	0.200±0.03	0.181±0.05	0.02
F4–15 Hz	0.192±0.03	0.173±0.05	0.001
PZ–15 Hz	0.203±0.03	0.174±0.04	0.001
P3–15 Hz	0.210±0.03	0.184±0.05	0.005
P4–15 Hz	0.199±0.03	0.171±0.04	0.001

Table 3 shows the mean spatial complexity ± standard deviation and associated p values for those EEG electrodes that were statistically significant between outcome groups, using the Bonferroni correction for multiple comparisons (p value of <0.003 were statistically significant). Patients with good outcome had higher spatial complexity in the frontal – parietal electrodes compared to those with poor outcome.

## Discussion

The results of our analysis indicate that children admitted to the PCCU in coma with brain injury secondary to cardiac arrest, TBI or stroke who had a poor outcome (PCPC score 4 to 6) had a higher magnitude of phase synchrony (R index), lower spatial complexity of the synchrony patterns and lower temporal variability of the R index values at 15 Hz when compared to those patients with a good outcome (PCPC score 1 to 3). Thus, these results indicate that there is an association between the severity of the brain injury and the spatio-temporal variability of the synchronization patterns. The mean global R indices were significantly higher and the spatial complexity and temporal variability values were significantly lower at the beta frequency in patients with poor outcome. We also showed that patients with poor outcome had lower spatial complexity of EEG synchrony at 15 Hz in the frontal and parietal EEG electrodes as compared to those patients with a good outcome.

Based on the known neurophysiological activity following TBI, it is conceivable that there will be fundamental alterations in the brain coordination dynamics, reflected as synchronization patterns. For instance, neuronal hyper-excitability in early post-traumatic periods has been demonstrated both in vitro and in vivo [31]. Following cortical injury there are time dependent alterations in synaptic function and cell reorganization and damage [32], [8]. Electrophysiological analysis of cerebral concussion has had a long history [43]. Earlier studies evaluated EEG coherence in children and alterations in the coherence patterns were thought to reflect neuroanatomical inhomogeneities corresponding to features of neocortical cytoarchitecture and axonal fibre systems [46], [47]. Thatcher et al. (1989) proposed that the analysis of coherence in post-traumatic EEG activity can detect and quantify diffuse axonal injury [48], [49]. However, very few studies have addressed this line of research in post-traumatic brain injury, but it has been investigated in depth in other pathologies.

The use of EEG phase synchrony in brain injury is more recent. The study by Shields et al. (2007) done in adults, reported a global decrease in synchrony as patients emerged from coma, which correlated with the Glasgow Coma Scores for the individual subjects [51]. Their findings supported those of our initial paper, where children emerging from coma had increased variability and decreased prolonged EEG phase synchronization.

Recently EEG phase synchrony has been used to evaluate brain function of adults following stroke and cardiac arrest [71]–[73]. Wu et al., (2011) found that focal injury following stroke in elderly patients resulted in breakdown of cortical synchrony networks, interrupting large scale communication [71]. Functional brain networks are altered in adult stroke patients [72]. Cimponeriu et al., (2002) found changes to phase synchronization in the theta range in the first hour post cardiac arrest [73]. We cannot directly compare EEG findings between adults and children. However the alterations in brain networks are seen in children post brain injury [34]. The findings in the adult studies could reflect our finding of significant phase synchrony and variability changes post injury in the 15 Hz beta bandwidth. The EEG phase synchrony in the beta range is associated with long range connectivity among brain regions beyond that of local neighbouring neuronal networks [74]. As brain injury often involves white matter damage, the structural pathway between neurons would be disrupted. This in turn would change the EEG phase synchronization in the beta range. The beta frequency has also been associated with normal functioning in the motor and somatosensory cortices and represents the intrinsic oscillations in the thalamocortical circuits in these two brain regions [75], [76]. The thalamocortical circuitry is disrupted in the comatose patient [77].

In general, the analysis of synchrony has potentially more information than other more classical studies, such as power spectra determination, as the latter does not inform on coordinated brain activity [34], [67]. Phase synchrony provides the added information of connectivity among EEG electrodes, as electrodes that are highly phase-locked are connected for that time epoch [67]. In this regard, Davey et al. (2000) demonstrated that, while power spectra did not differ from the two brain hemispheres of a patient with asymmetric brain damage (one hemisphere was damaged whereas the other was not), the coherence of the recorded signals was markedly different between the two hemispheres, thus stressing the importance to assess not only whether the brain rhythms have been normalised after brain damage (which is analysed using power spectra), but also to study the coordinated activity between distant brain areas using coherence of phase synchrony methods[78].

Analytical methods that evaluate neuronal synchronization among brain areas provide more information than visual evaluation of the electroencephalogram alone, and thus could be of importance in clinical settings. In particular, phase synchrony analysis based on the analytic signal approach has been used to assess aspects of brain coordination dynamics [74], [79]. Normal brain function is thought to result from fluctuating patterns of synchronization and desynchronization between cell networks. These fluctuations are a reflection of the information processing occurring in the brain networks [74], [80]. Hence, it is not surprising that less variability in brain signals is associated with unconscious states or pathologies in general [81], [82]. As presented in the introduction, physiological variability is associated with healthy conditions, as it is very well-known in the cardiac field, for example, where lower variability in heart activity is associated with cardiac injury [83], [84]. We and others, (Garrett et al., 2011, 2013) propose that a similar trend occurs in brain function: less variability in the fluctuating patterns of brain activity will be associated with brain dysfunction [85], [86]. While the patterns of phase synchronization changes may be different based on the causative factor (TBI, stroke or cardiac arrest), injury will result in less variability of EEG phase synchronization. Our previous pilot study on synchronization of EEG signals after TBI used a few patients and control participants, and the results indicated that patients had lower spatio-temporal variability in the synchrony patterns than age-matched control subjects [34]. In this study, we have obtained a larger set of patients and correlated the variability of the EEG synchrony to the clinical outcome. Using other variability measures (multiscale entropy) Raja Beharelle et al. (2012) reported lower variability derived from magnetoencephalographic (MEG) recordings in TBI patients performing an attention task, observations that indicate that the tendency towards lower fluctuation in brain signals may be a general phenomenon after brain injury, and not only occurring in the acute post-traumatic phase we studied here [87]. Recently a complexity analysis of resting state MEG activity in adult soldiers who had suffered mild TBI found that patients had lower variability compared to control subjects [88]. Further, those patients who had higher variability recovered the most cognitive ability.

While global measures of EEG phase synchrony and spatio-temporal variability show promise as markers of brain function, we were also interested in which electrodes were individually significant. If the EEG synchrony and variability are to be used broadly for patient monitoring, we have to consider common hospital constraints. For instances when, availability of nursing staff, availability of EEG technologists, or presence of patient instrumentation (drains, monitors) precluded the application of the 10–20 montage, we were interested in evaluating which electrodes may yield the best discrimination.

We found that the spatial complexity of fronto-parietal and midline parietal electrodes were significantly higher in those patients with a good outcome. Interpretation of this finding is difficult as there are no comparable EEG phase synchrony studies. We looked for comparisons by extrapolating from neuroimaging studies. Positron Emission Tomography (PET) scans evaluating cerebral glucose metabolism have shown that fronto-parietal connectivity is important for awareness and that decreased connectivity in these regions is associated with coma and anaesthesia [89]–[90]. Functional MRI (fMRI) has also been used to show that in coma and minimally conscious states there is impaired connectivity among fronto-parietal regions of the default network of the brain [91], [92]. This finding warrants further study. In future we would evaluate an abbreviated montage for monitoring patients by utilizing frontal and parietal electrodes.

All of these findings, both global and electrode specific must be considered within the limitations of a retrospective study. Timing of the EEGs in relation to the event varied as there was no protocol for when to order EEGs in any of the diagnostic categories. The EEGs were ordered when clinicians’ observations indicated that the patient may be having clinical seizures or when subclinical seizures were thought to be the cause of the coma. Thus the EEG and its phase synchrony and spatio-temporal variability would reflect the child’s brain function at the time of recording. If the brain injury was severe enough to lead to poor outcome, this should be reflected in high R index values and low spatio-temporal variability. The long term goal is to make these indices derived from continuous EEG available for real-time brain monitoring as opposed to one point in time, enabling clinicians to trend R index changes over time.

The same is true of the other challenge of this retrospective study: the heterogeneity of medications used in treating the patients. Again for our proposed biomarker of brain synchrony and variability to be clinically useful, it must reflect the patient’s brain activity as it is affected by both injury and medication. Prolonged sedation adversely affects patient outcome [93]. Whether the need for prolonged sedation reflects the patient’s overall condition that negatively impacts the brain leading to poor outcome or whether sedation has augmenting adverse effects on the injured brain, the R index and its variability should reflect this. As our findings warrant further study, the effects of medication will be evaluated when these indices can be monitored by clinicians in real-time. Not only will the effect of the addition or titration of medication on the R index be able to be evaluated, but changes in ventilator settings, arterial pressure and intracranial pressure will be tracked.

## Conclusion

The results of this study supported our hypothesis that comatose paediatric patients who had a good outcome would have lower EEG phase synchrony and higher spatio-temporal variability than those patients with poor outcome. Our methodology could be very useful in the prediction of outcome in paediatric patients with brain injury during the acute phase post-injury. We propose that variability of EEG phase synchrony will become a tool that provides brain function monitoring and can correlate with outcomein critically ill comatose patients.
